# Supplementary material for: No increased circular inference in adults with high levels of autistic traits or autism
Source: PLoS Comput Biol. 2021 Sep 24;17(9):e1009006. doi: 10.1371/journal.pcbi.1009006 (PMC8494311; doi:10.1371/journal.pcbi.1009006)
Supplement: S1 Supplementary Information — Table B3 in S1 Supplementary Information. Kendall rank correlations between recovered CINI parameters. Table B4 in S1 Supplementary Information. Kendall rank correlations between recovered CII parameters. Fig D1 in S1 Supplementary Information. CINI (top) and CII (bottom) model fit vs participant logit confidence estimates. Fig E1 in S1 Supplementary Information. Reverberation parameters of the current study’s ASD sample and Jardri et al.’s SCZ sample. Table E3 in S1 Supplementary Information. Fixed and random effects model comparisons in both studies. (PDF) [file pcbi.1009006.s001.pdf]

No increased circular inference in adults with high levels of autistic traits or autism:

S1 Supplementary Information

N. Angeletos Chrysaitis, R. Jardri, S. Denève, and P. Seriès

A. Data collection and processing

1. Experiment details and pre-screening

The platform Prolific was chosen for its higher data quality compared to the alternatives [1]. Both Prolific and social media participants filled in the questionnaires before they participated in the behavioural task. We included two attention checks in the AQ questionnaire (i.e., questions that straightforwardly asked for a specific response) to serve as validity checks for data quality. The task was implemented on the PsychoPy 2020.1.3 Builder, automatically translated to PsychoJS, and hosted on Pavlovia [2], as this method achieves high temporal accuracy [3].

Prolific asks its participants various questions, which we used to pre-screen our participants. We required all participants to have answered positively to the question ‘Do you have normal or corrected-to-normal vision? (i.e., You can see colour normally, and if you need glasses, you are wearing them or contact lenses)’ and negatively to the question ‘Are you currently taking any medication to treat symptoms of depression, anxiety or low-mood (e.g., SSRIs)?’.

Moreover, we used the question ‘Have you received a formal clinical diagnosis of autistic spectrum disorder, made by a psychiatrist, psychologist, or other qualified medical specialist? This includes Asperger's syndrome, Autistic Disorder, High Functioning Autism or Pervasive Developmental Disorder’ to ensure that we would get a broad enough range for autistic traits. The question had the following possible answers:

1. Yes – as a child
2. Yes – as an adult
3. I am in the process of receiving a diagnosis
4. No – but I identify as being on the autistic spectrum

5. No
6. Don't know / rather not say

We recruited half of our Prolific participants from those that had chosen options 1-4, and half from those that had chosen options 5 or 6. All participants had taken part in at least 3 more studies, with an approval rate of at least 98% (meaning that their submissions in previous studies have been approved 98% of the time).

2. Trial set

Trials where priors and likelihoods were both very high or very low were not included in our trial set, as participants would presumably respond to the extremes of the scale, not providing much information about the integration of priors and likelihoods. Instead, we focused on trials with stimuli closer to a probability of 0.5 (equal basket sizes or fish proportions), so that prior and likelihood overcounting would be more apparent. Trials with stimuli equal to 0.5 were avoided, as, independently of parameter values, stimuli equal to 0.5 do not influence the model estimates. The resulting trial set is presented in Table A1.

Table A1. Counts of prior-likelihood combinations in the trial set.

		Likelihood values								
		0.1	0.2	0.3	0.4	0.5	0.6	0.7	0.8	0.9
Prior values	0.1	–	–	2	2	1	2	2	1	1
	0.2	–	1	2	2	1	2	2	1	1
	0.3	2	2	2	2	2	2	2	2	2
	0.4	2	2	2	2	2	2	2	2	2
	0.5	1	1	2	2	–	2	2	1	1
	0.6	2	2	2	2	2	2	2	2	2
	0.7	2	2	2	2	2	2	2	2	2
	0.8	1	1	2	2	1	2	2	1	–
	0.9	1	1	2	2	1	2	2	–	–

3. Data cleaning

Crowdsourcing data for behavioural experiments online carries many risks in terms of data quality. Participants might misunderstand the instructions, be prone to external distractions, view the experiment only as a source of income so that they aim to finish it as fast as possible at the expense of quality, or even use automated programs (bots) to complete it [4]. To filter out these risks, various measures of data quality have to be implemented. In this experiment, as detailed below, we used four criteria to help us distinguish between high- and low-quality responses: the attention checks, the responses to the more ‘certain’ trials of the task, the distributions of task responses, and the average time per response. All of them were chosen and implemented before the data were analysed in any other way.

Specifically, for the attention checks, all participants who failed at least one were immediately disqualified (n = 9). ‘Certain’ trials are those with very high (> 0.94) or very low (< 0.06) Bayesian posteriors, i.e., those with a (*prior, likelihood*) combination of (0.7, 0.9), (0.9, 0.7), (0.8, 0.8), (0.3, 0.1), (0.1, 0.3), and (0.2, 0.2). There was a total of 16 such trials, including the last 6 training ones. We discarded all participants who clicked on the opposite side of the scale relative to the posterior in more than 3 out of these 16 trials (n = 3), as they could not have been using the information provided to make a choice. Then, we disqualified the participants who mostly clicked outside of the scale (n = 1), and those who had clustered responses (n = 9), as they seemed to have discretized the scale instead of treating it as a continuous measure of confidence (Fig A1). Finally, we discarded data from participants who had an average response time of more than 5*s* (n = 6), because it might signify alternative response strategies compared to the other participants, given that we had instructed them to answer ‘as fast and precise as possible’. Data were discarded slightly more often from the ASD population (Table A2). The final sample showed good reliability for the total AQ (*Cronbach’s a* = 0.76) and slightly low reliability for the PDI Y/N (*Cronbach’s a* = 0.67).

Table A2. Number of discarded participants for each Prolific ASD category.

ASD category	*Total sample*	*Discarded*	*Proportion*
1	19	3	0.16
2	7	2	0.29
3	5	1	0.2
4	39	5	0.13
5	68	7	0.1
6	2	0	0
– (social media)	61	7	0.11

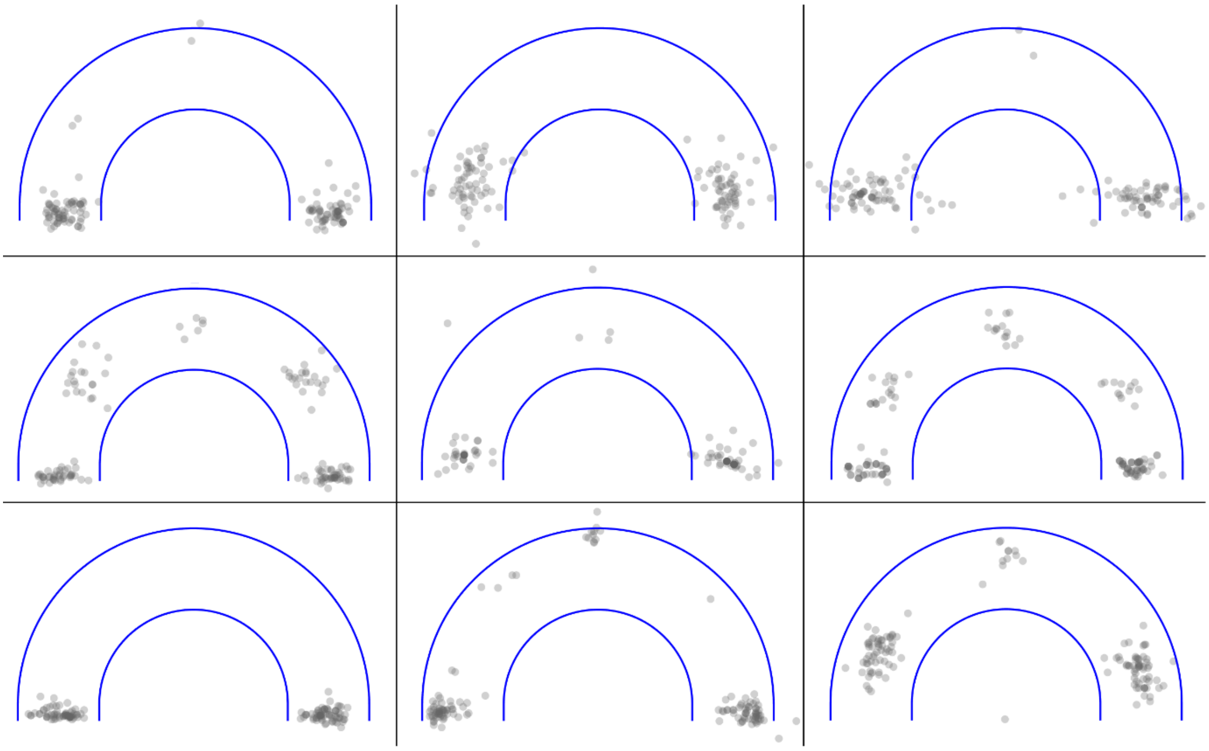

Fig A1. The 9 datasets that got discarded because of response clustering.

Blue lines represent the edges of the response scale and grey dots the locations of the mouse clicks by the participants. The presented participants seem to have misinterpreted the instructions of the task, answering in a discrete instead of a continuous way, in 2, 3, or 5 distinct places.

4. Statistical Power

Jardri et al. found a Pearson's correlation of 0.59 between sensory evidence reverberation and non-clinical delusionary beliefs in their whole sample, as well as a correlation of 0.45 between sensory evidence reverberation and psychotic symptoms in their SCZ subsample. With a significance level of $a = 0.05$ and our sample size $n = 176$, a Kendall's correlation coefficient of only $\tau = 0.2$ yields a statistical power of 98%, as calculated based on hypothesis testing [5]. It is important to note that all power calculations for Kendall's τ assume bivariate normality, which is not verified in our dataset. Therefore, this value should be understood only as a weak indication of the actual power of our correlation tests. Similarly, the statistical power for the group comparisons cannot be calculated without any assumptions for the underlying distribution. However, group sizes in the present study are comparable to those used by Jardri et al., who showed very clear effects.

B. Modelling details

1. Linear mixed-effects models

The linear mixed-effects models (LMEs) that were used in the current study are shown below in Wilkinson-Rogers notation. `absCnf` stands for absolute confidence, `absLl` for absolute likelihood, `prCng` for prior congruency, `RT` for the reaction times, and `ID` for the participant ID. For an analysis of the role of each model component, please see the corresponding Methods and Materials section in the main text.

LME_core:

```
absCnf ~ absLl*prCng + RT + (1|ID)
```

LME_AQ:

```
absCnf ~ absLl*prCng*AQ + RT + (1|ID)
```

LME_PDI:

```
absCnf ~ absLl*prCng*PDI + RT + (1|ID)
```

LME_full:

```
absCnf ~ absLl*prCng*AQ + absLl*prCng*PDI + RT + (1|ID)
```

LME_rtInteract:

```
absCnf ~ absLl*prCng*AQ + absLl*prCng*PDI + RT*AQ + RT*PDI + (1|ID)
```

The best BIC score was achieved by LME_core (Fig B1), showing that AQ and PDI scores offered relatively little information. Despite that, the full results for all models can be seen in Table B1.

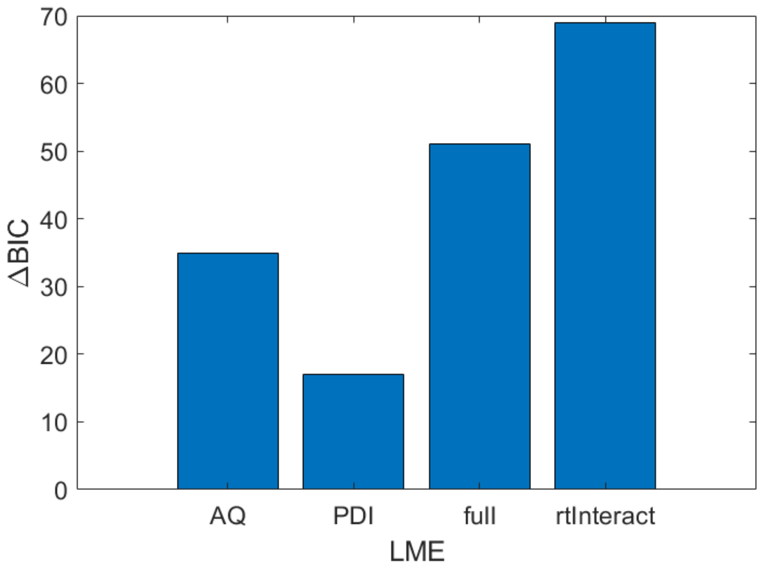

Fig B1. Results of model comparisons.

ΔBIC is the difference between the BIC score of each model and that of LME_core. Lower BIC scores are better, with differences of 20 or more being considered very strong evidence [6].

Table B1. Results of linear mixed-effects models.

Model	Component	*t*	*p*	Component	*t*	*p*
LME_core	`absLl`	44.50	$<10^{-323}$	`absLl:prCng`	25.20	10^{-138}
	`prCng`	24.63	10^{-132}	`RT`	–17.01	10^{-64}
LME_AQ	`absLl`	11.04	10^{-28}	`AQ`	–1.72	0.09
	`prCng`	8.09	10^{-16}	`absLl:AQ`	1.23	0.22
	`absLl:prCng`	5.46	10^{-8}	`prCng:AQ`	–1.39	0.16
	`RT`	–17.03	10^{-64}	`absLl:prCng:AQ`	1.52	0.13
LME_PDI	`absLl`	18.19	10^{-73}	`PDI`	2.08	0.04
	`prCng`	10.83	10^{-27}	`absLl:PDI`	2.31	0.02
	`absLl:prCng`	12.84	10^{-37}	`prCng:PDI`	0.40	0.69
	`RT`	–17.00	10^{-64}	`absLl:prCng:PDI`	–1.55	0.12
LME_full	`absLl`	9.03	10^{-19}	`absLl:AQ`	1.08	0.28
	`prCng`	7.10	10^{-12}	`prCng:AQ`	–1.42	0.15
	`absLl:prCng`	5.65	10^{-8}	`absLl:prCng:AQ`	1.63	0.10
	`RT`	–17.01	10^{-64}	`absLl:PDI`	2.23	0.03
	`AQ`	–1.91	0.06	`prCng:PDI`	0.50	0.62
	`PDI`	2.23	0.03	`absLl:prCng:PDI`	–1.66	0.1
LME_rtInteract	`absLl`	8.90	10^{-18}	`PDI`	1.88	0.06
	`prCng`	7.06	10^{-12}	`absLl:PDI`	2.32	0.02
	`absLl:prCng`	5.55	10^{-8}	`prCng:PDI`	0.55	0.58
	`AQ`	–2.03	0.04	`absLl:prCng:PDI`	–1.58	0.11
	`absLl:AQ`	1.14	0.25	`RT`	–5.08	10^{-7}
	`prCng:AQ`	–1.40	0.16	`RT:AQ`	0.72	0.47
	`absLl:prCng:AQ`	1.68	0.09	`RT:PDI`	1.29	0.20

Model components with $p < 0.05$ *are shaded.*

2. Bayesian models

All the following procedures were identical to those reported by Jardri et al. [7].

Participant and model confidence estimates were restricted to the range [0.01, 0.99], to avoid numerical issues in both the reporting of participant responses and the model fitting. Moreover, trials where participants did not click on or very close to the scale were not included in our analysis (see the code at https://osf.io/yqug2/ for the exact criterion).

During model fitting, we applied a small L2 regularization penalty on the reverberation parameters (a) to prevent 'degenerate' solutions with weights close to 0 and artificially large a. This penalty was equal to $0.00005(a_p^2 + a_s^2)$. Model predictions were almost completely insensitive to this added cost. Due to the regularization, reverberation parameters rarely exceeded a value of 0.5, as the minuscule benefit to the model predictions (Fig B2) was outweighed by the L2 penalty. Model fitting was carried out by minimizing mean squared error, which is equivalent to least squares. This was chosen so that the L2 term would not change depending on the number of trials included for each participant.

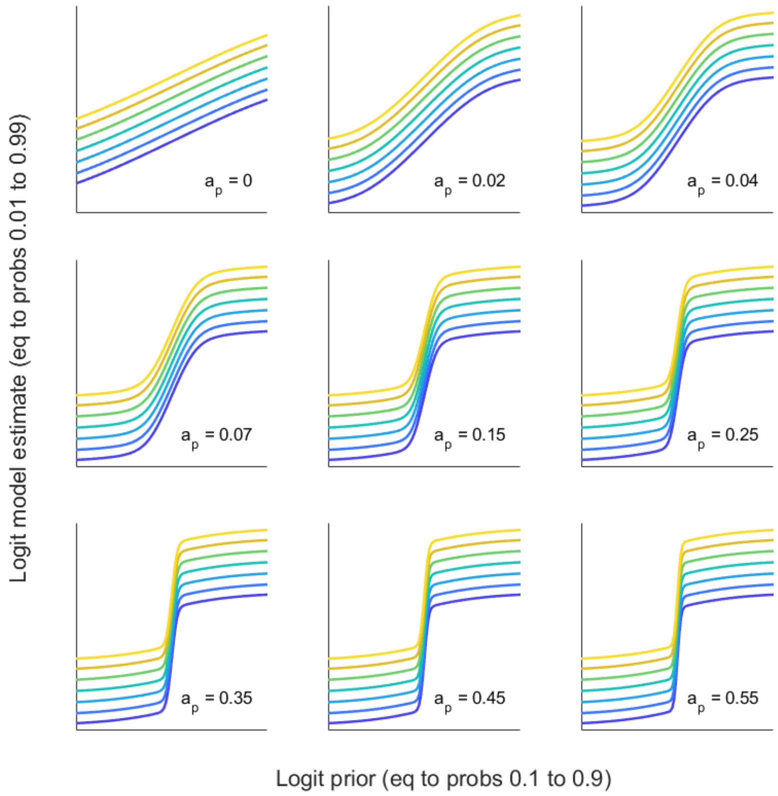

Fig B2. *CINI* predictions with varying prior reverberation.

Coloured lines correspond to different likelihood values (probabilities 0.1 to 0.9). Changes from $a_p = 0$ *(top left) to* $a_p = 0.15$ *(middle) greatly affect observed model predictions, while those from* $a_p = 0.15$ *to* $a_p = 0.55$ *(bottom right) have almost no perceptible effect, despite the larger difference between parameter values.*

3. Parameter Recovery

First, for each selected model, we created 1000 simulated participants by drawing values randomly from the set of estimated parameters and adding a small uniform noise term drawn from [–0.025, 0.025]. Each simulated subject was also assigned an error variance, calculated from a random participant's mean squared error around the model estimate, L_c. Then, we generated 130 trial

responses for each simulated subject using a Gaussian distribution with the assigned variance, centred on the model response. Finally, both models were fitted on the simulated responses, and the recovered parameters were compared with the original ones using Pearson's product-moment correlation coefficient. We also tested for correlated parameters, by calculating the Kendall rank correlation between the recovered parameter values. Model recovery was performed by fitting the simulated responses of all participants with both models and calculating the confusion matrix.

Most parameters were recovered close to their original values for both CINI (Fig B3) and CII (Fig B4). However, reverberation parameters were much more likely to be badly recovered if they had values greater than 0.15, leading to a clustering pattern (top-left plot of Fig B3 and B4). In this range, the data can be roughly approximated by treating prior or likelihood information as binary (e.g., 'left basket larger' vs 'right basket larger'), with very little dependency on the exact size of the baskets or the exact fish ratios (Fig B2). However, this was a minority of the estimated values.

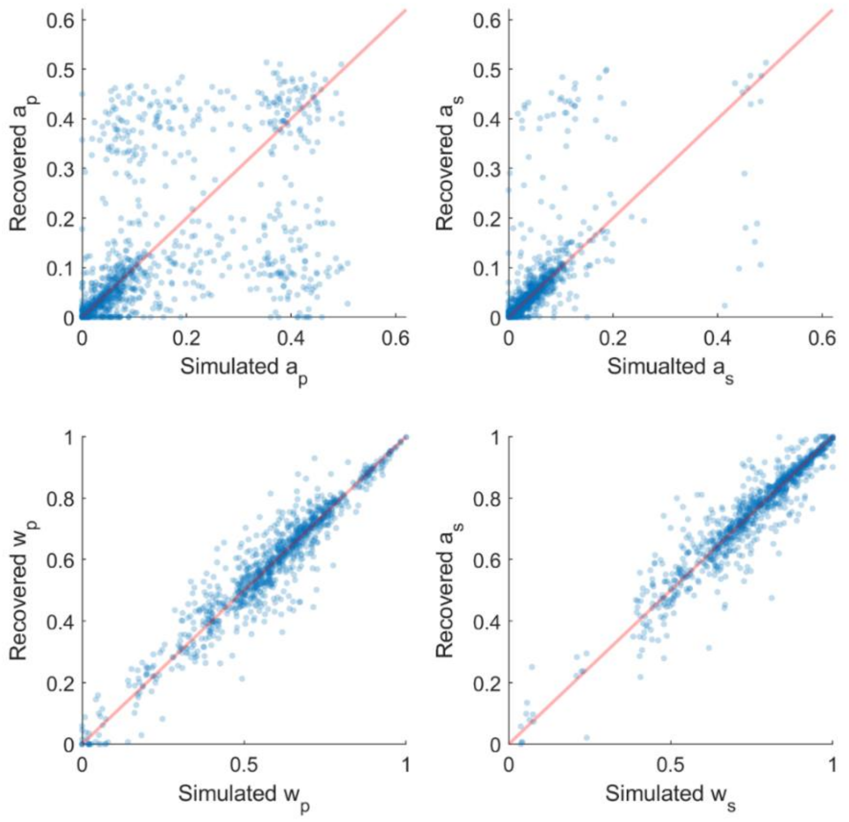

Fig B3. Recovered vs simulated parameters of CINI model.

Each dot represents one simulated participant. The red line represents perfect recovery.

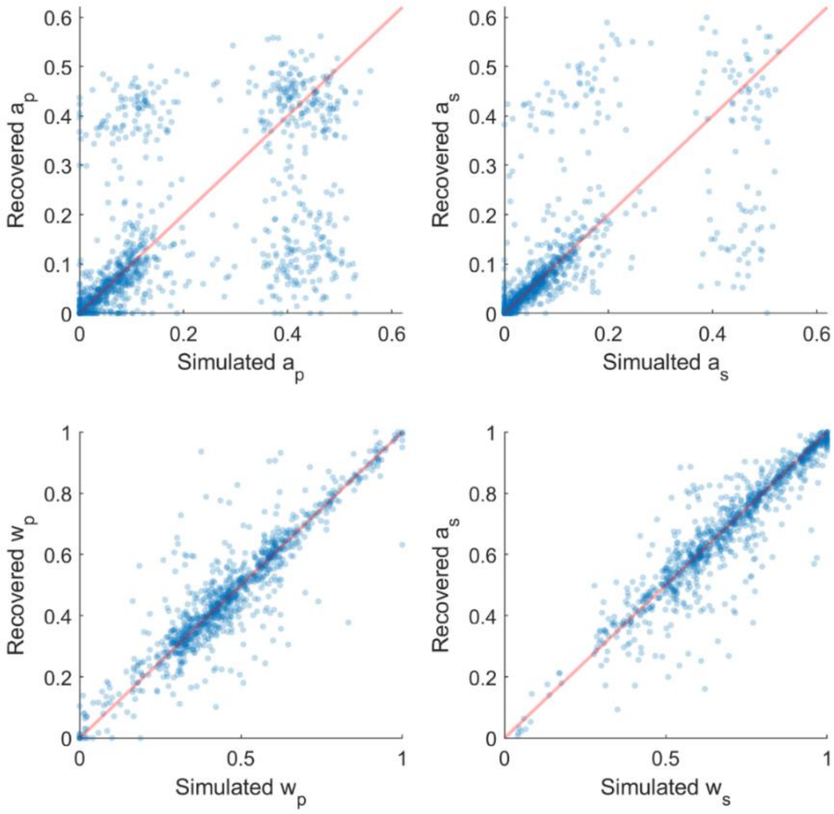

Fig B4. Recovered vs simulated parameters of CII model.

Each dot represents one simulated participant. The red line represents perfect recovery.

The clustering pattern can also be observed when parameter recovery used the set of 200 trials of Jardri et al. [7] (Fig B5), with minimal differences in the recovery correlations between the two sets (Table B2).

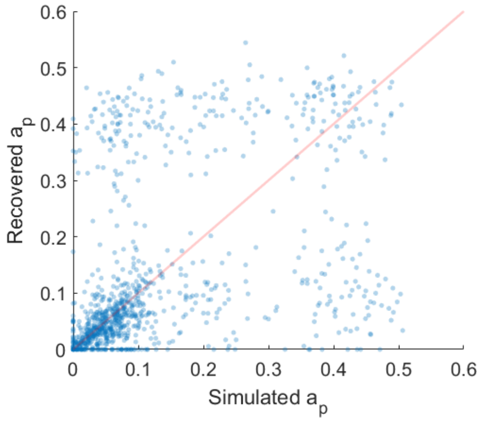

Fig B5. Parameter recovery results for the *CINI* prior reverberation over 200 trials. *The red line corresponds to perfect recovery.*

	CINI		*CII*	
**Trials**	**130**	**200**	**130**	**200**
a_p	0.54	0.46	0.54	0.51
a_s	0.58	0.54	0.71	0.60
w_p	0.96	0.95	0.94	0.92
w_s	0.91	0.94	0.93	0.93

Table B2. Pearson correlations between simulated and recovered parameters in the trial set of Jardri et al. and the current study.

Minimal correlations were observed between the recovered parameters of both the CINI (Table B3) and CII model (Table B4). The same pattern of minimal correlations was also observed between the simulated and recovered values across parameters (all | τ | < 0.1).

Table B3. Kendall rank correlations between recovered CINI parameters.

a_p	1			
a_s	–0.012	1		
w_p	–0.008	–0.022	1	
w_s	0.016	–0.043	–0.041	1
	a_p	a_s	w_p	w_s

Table B4. Kendall rank correlations between recovered CII parameters.

a_p	1			
a_s	–0.019	1		
w_p	0.040	–0.037	1	
w_s	0.007	–0.020	–0.083	1
	a_p	a_s	w_p	w_s

C. Visualizations

1. Model parameters and correlations with AQ

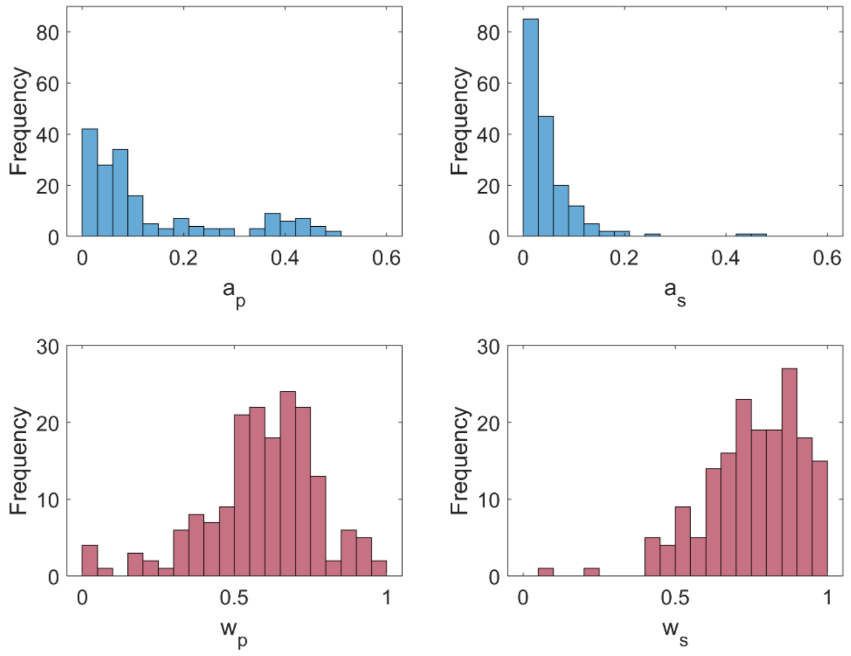

Fig C1. Histogram of CINI parameter values estimated during model fitting.

Notice that axis limits differ between rows.

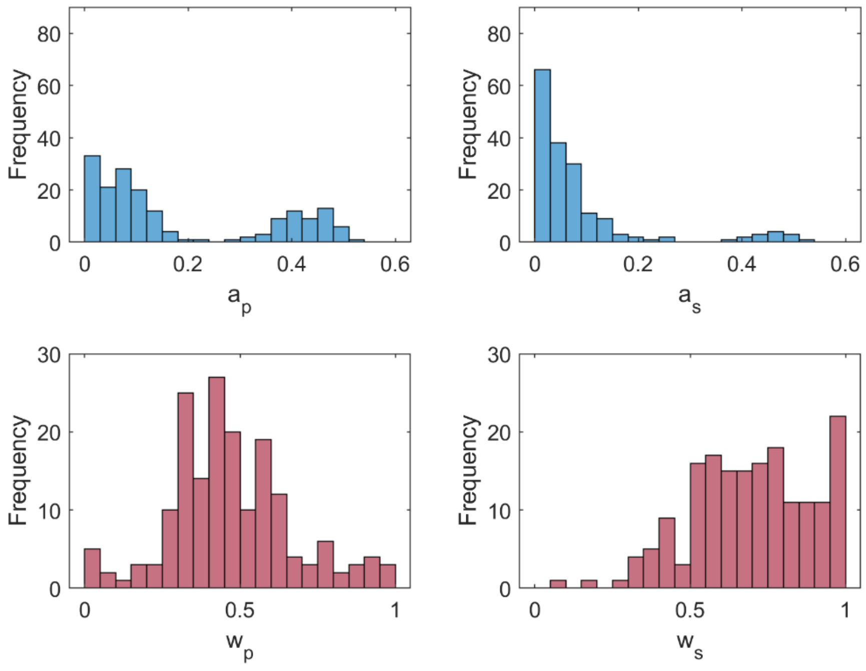

Fig C2. Histogram of CII parameter values estimated during model fitting.

Notice that axis limits differ between rows.

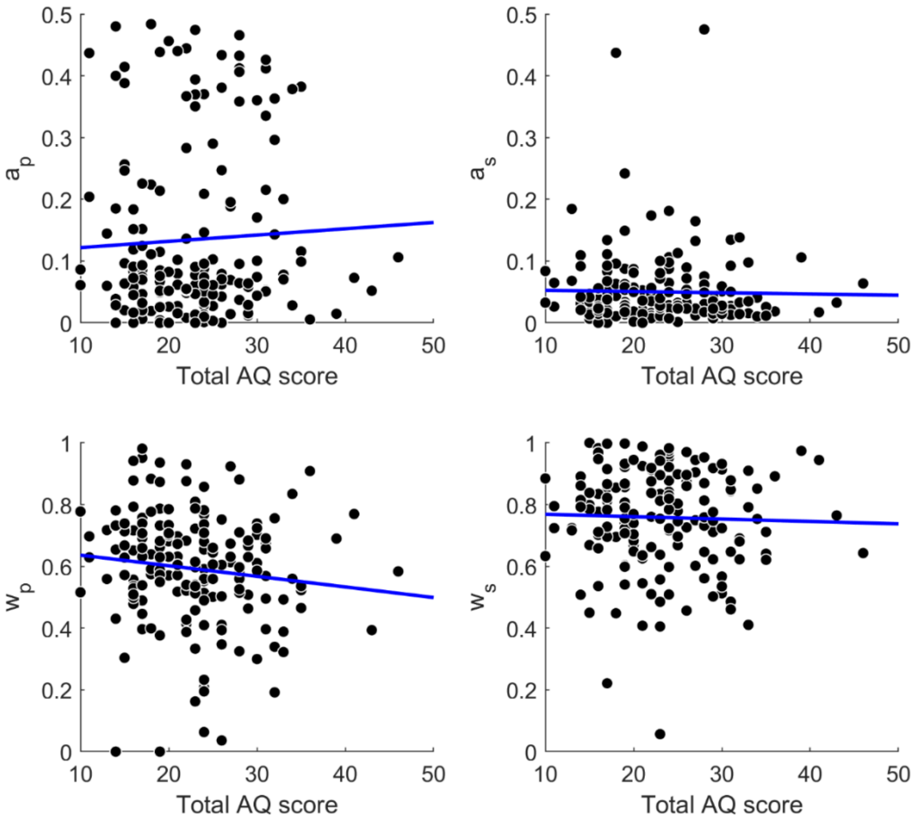

Fig C3. CINI model fit parameters vs participant AQ.

The blue line represents the least squares fit. All of the presented correlations had an uncorrected p-value above 0.05, besides w_p *(*$\tau = -0.12$*,* $p = 0.02$*), that however would not survive correction for multiple comparisons.*

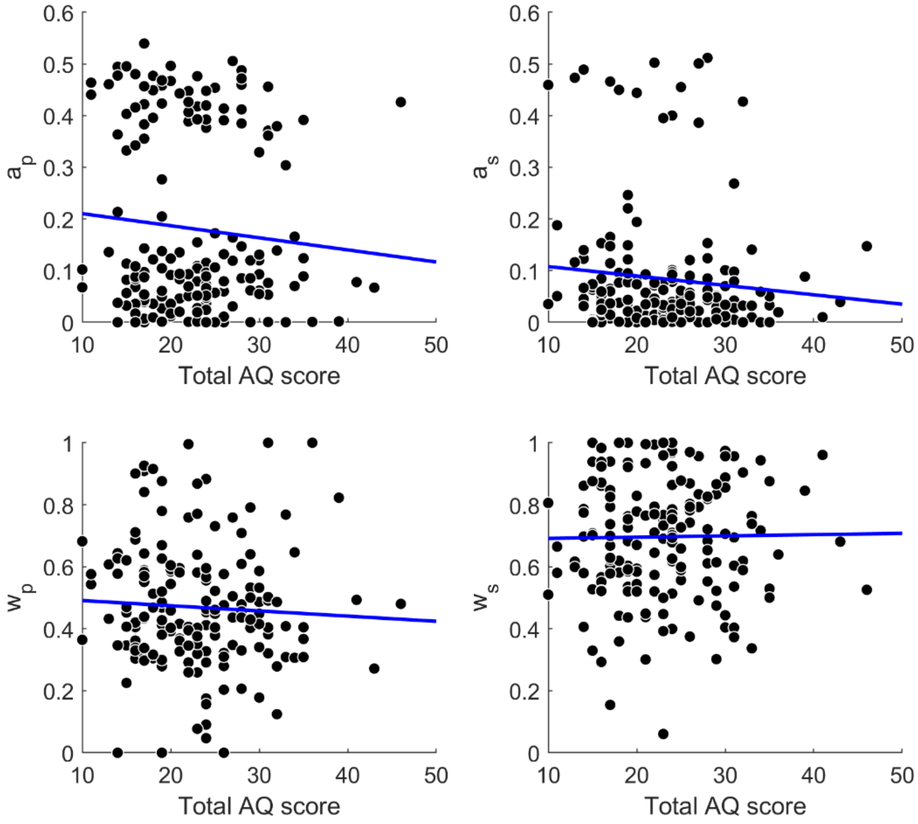

Fig C4. CII model fit parameters vs participant AQ.

The blue line represents the least squares fit. All of the presented correlations had an uncorrected p-value above 0.05.

D. Additional tests

1. ASD vs ND with low AQ

To avoid comparing autistic participants to participants with a potential undiagnosed autism spectrum disorder, we performed an additional comparison with a limited ND group, which contained only participants with AQ scores lower than or equal to the reported mean value of the general population [8] (AQ ≤ 17, n = 21). The results showed no differences between the groups (Table D1).

Table D1. Mann-Whitney U test results between the CINI model fit parameters of the ASD and limited-ND participant groups.

	**limited-ND vs ASD**		
**CINI params**	f	p	BF_{01}
$\boldsymbol{a_p}$	0.51	0.90	3.26
$\boldsymbol{a_s}$	0.44	0.50	2.52
$\boldsymbol{w_p}$	0.46	0.63	2.88
$\boldsymbol{w_s}$	0.57	0.44	2.61

f signifies the common language effect size, with larger f values corresponding to larger parameter values for the ASD group, relative to the limited-ND. p-values are uncorrected for multiple comparisons.

2. Circular Inference – Interference

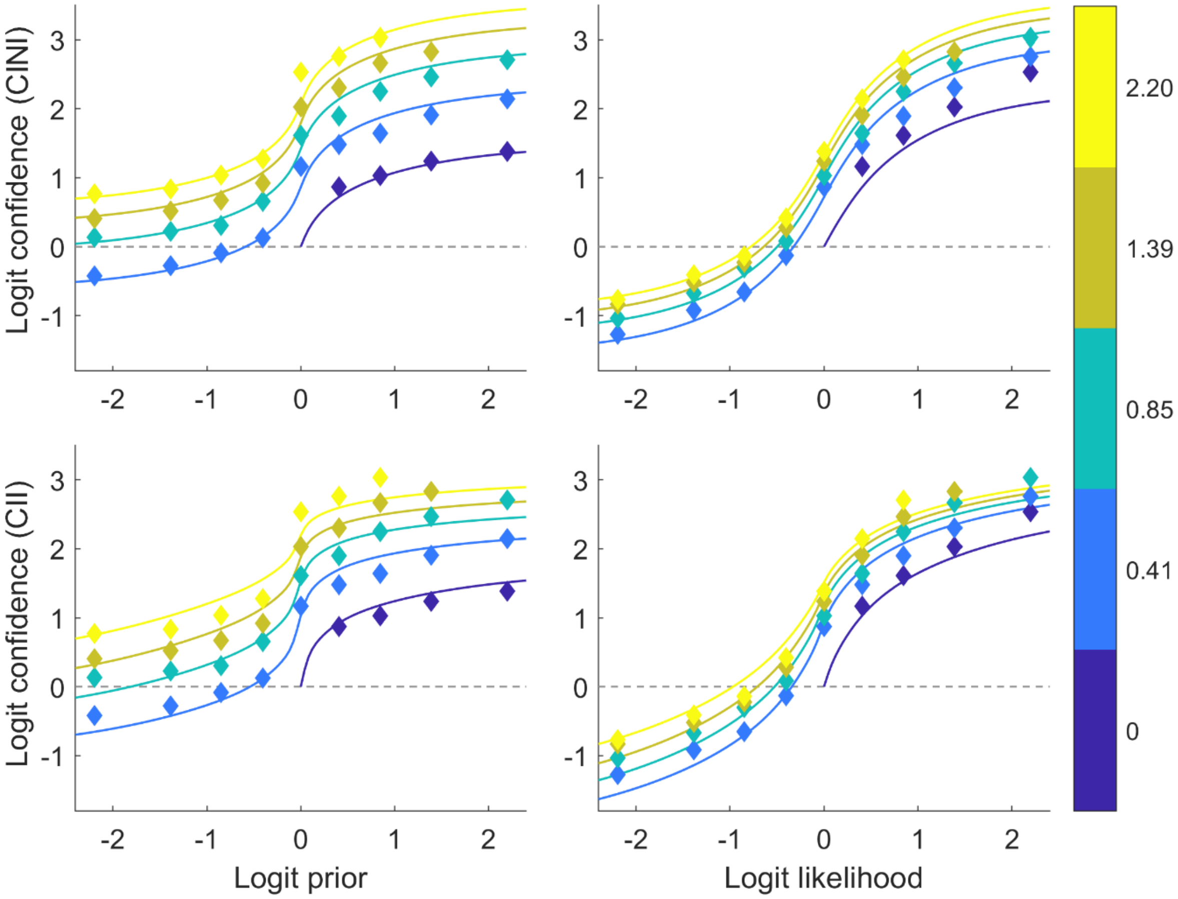

Fig D1. CINI (top) and CII (bottom) model fit vs participant logit confidence estimates. *Model and participant logit confidence as function of logit likelihoods and priors. Coloured lines represent model predictions and rhombuses the subject confidence estimates. Different colours represent logit likelihood values in the left graph and logit prior values in the right and are equivalent to probabilities 0.5 to 0.9. Since both the task and the two model structures are symmetrical around 0 logit confidence (0.5 probability), participant estimates have been averaged between symmetric trials to reduce noise (e.g., a trial with a logit prior of –1 and a logit likelihood of 2 would have been symmetrical to one with a logit prior of 1 and a logit likelihood of –2).*

Kendall correlations showed no association between AQ and any CII parameters (Table D2). Moreover, no differences were found in model parameters between the ND and ASD groups (Table D3, Fig D4). Pearson correlations between CII and CINI parameters were high (a_p, r = 0.75; a_s, r = 0.76; w_p, r = 0.82; w_s, r = 0.83).

Table D2. Kendall rank correlations between CII parameters and psychiatric traits.

	AQ			PDI		
**CII params**	τ	p	BF_{01}	τ	p	BF_{01}
a_p	–0.05	0.38	6.84	–0.05	0.34	6.19
a_s	–0.08	0.14	3.23	0.00	0.94	10.1
w_p	–0.07	0.18	3.95	0.08	0.14	3.19
w_s	0.01	0.81	9.84	0.09	0.09	2.16

Total AQ scores and Y/N PDI scores were used for the correlations. τ signifies the correlation coefficient. p-values are presented without any correction for multiple comparisons.

Table D3. Mann-Whitney U test results between the CII parameters of the ASD and ND groups and the low-AQ and high-AQ groups.

	ND vs ASD			high-AQ vs low-AQ		
**CII params**	f	p	BF_{01}	f	p	BF_{01}
a_p	0.55	0.50	3.82	0.44	0.40	2.37
a_s	0.45	0.46	3.15	0.40	0.18	1.81
w_p	0.47	0.64	3.85	0.43	0.36	3.14
w_s	0.53	0.71	3.71	0.49	0.93	3.77

Total AQ scores were used for the comparisons. f signifies the common language effect size, with larger f values corresponding to larger parameter values for the ASD and the high-AQ groups, relative to the others. p-values are uncorrected for multiple comparisons.

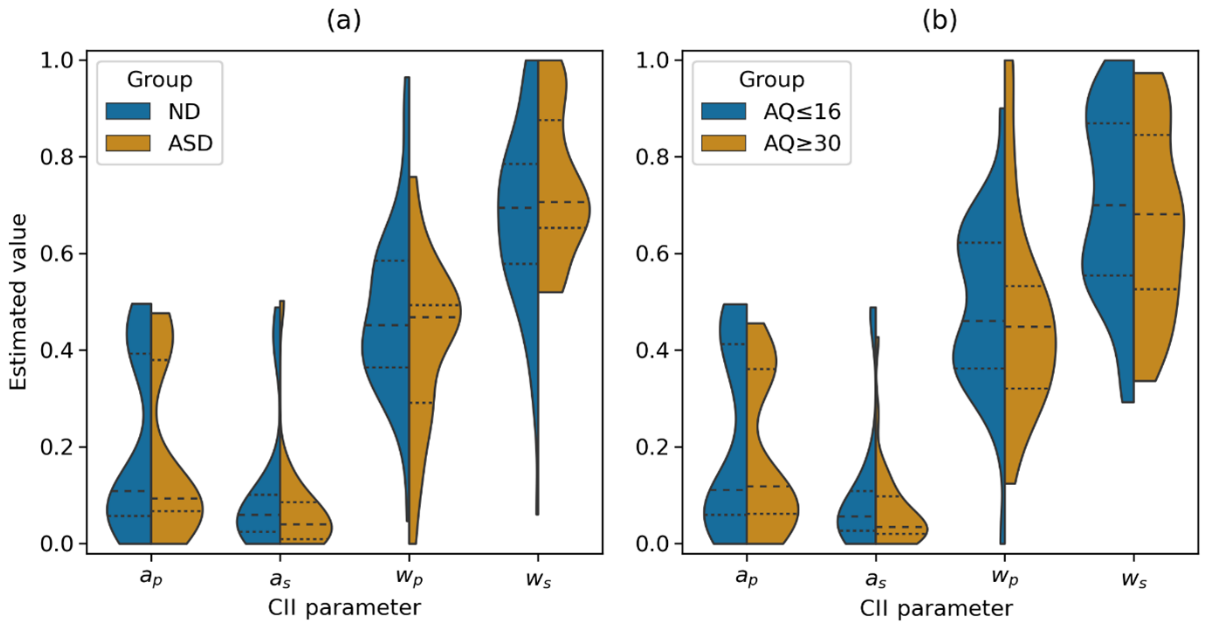

Fig D4. CII parameter values of ND vs ASD groups (a) and low-AQ vs high-AQ groups (b). *Violin plots show the density of estimated parameters over the possible values, relative to the group size. Dashed lines represent the median, while dotted ones represent the top and bottom quartiles in each group. No differences are observed between groups.*

3. AQ – Likert scoring

Scoring the AQ questionnaire on a Likert scale instead of the usual binary one (scores of 1-4 for each question, instead of 0 and 1), increased the questionnaire's reliability (*Cronbach's a* = 0.81), but did not affect the observed relationships with CINI model fit parameters. The Kendall correlation with the largest magnitude and the smallest corresponding *p*-value remained the one with the prior weights ($\tau = -0.12$, $p = 0.02$). As with the correlations reported in the main text, this relationship is non-significant when corrected for multiple comparisons. Moreover, once again, no comparisons were significant between the CINI model fit parameters of the new low- (Likert AQ ≤ 105, N = 28) and the new high- (Likert AQ ≥ 133, N = 29) AQ participant groups (e.g., w_p, uncorrected $p = 0.08$).

4. *Alternative interpretations of basket sizes*

Since the basket information is less quantitative than the fish ratios, we investigated whether it is interpreted by the participants as intended. For that, we tested four additional models that were based on CINI but accounted for different mappings between basket sizes and probabilities. The first model simply included a linear rescaling of the intended probabilities:

$$q_i = k(p_i - 0.5) + 0.5, \qquad i \in \{l, r\}, \qquad k \in (0, 1)\,, \tag{1}$$

with *p* corresponding to the intended probabilities, *q* to the perceived ones, *i* to the left or the right basket, and *k* to the rescaling factor. The second model allowed for the exponential rescaling of the probabilities:

$$q_i = \frac{p_i^k}{p_l^k + p_r^k}, \qquad i \in \{l, r\}, \qquad k \in (0, +\infty)\,, \tag{2}$$

The third was based on the Weber-Frechner law, under which the perceived sizes are proportional to the logarithm of the actual sizes [9]:

$$q_i = \frac{\ln\left(\frac{p_i}{p_0}\right)}{\ln\left(\frac{p_l}{p_0}\right) + \ln\left(\frac{p_r}{p_0}\right)}\,, \qquad i \in \{l, r\}, \qquad p_0 \in (0, 0.1)\,, \tag{3}$$

where $p0$ is the probability corresponding to the just noticeable basket size, assuming that the participants can see all the presented baskets. Finally, the fourth model treated basket sizes as providing only binary information ('left basket larger' vs 'right basket larger'), which nudged the fish frequency-based estimates by a constant amount. Then the logit confidence becomes:

$$L_c = F(L_s, w_s) \pm A, \qquad A \in (-\infty, +\infty)\,, \tag{4}$$

with A being the constant amount, positive when the left basket was larger than the right, negative in the opposite case, and equal to 0 when sizes were equal with each other.

The first three models had 5 parameters each, while the last one had only 3. We compared these models with the original CINI. Both fixed and random effects pairwise comparisons showed clear superiority for CINI, with group ΔBICs > 715 and posterior model probabilities for CINI > 0.72. This indicates that participants interpreted the basket sizes in the intended way of probabilities being analogous to basket size.

E. Comparisons between Jardri et al.'s and this study's datasets

To verify that the change of trial set did not have a strong influence on the parameter estimation, we compared the parameter values estimated from the original Jardri et al. dataset [7], with a subset of that dataset, restricted on the trials of the present study. Pearson's correlations showed minimal changes between parameters estimated from 200 and 130 trials (Table E1).

Table E1. Pearson's correlations between the parameters estimated from 200 and 130 trials of the Jardri et al. dataset.

CINI params	*R*	CII params	*R*
a_p	0.86	a_p	0.82
a_s	0.99	a_s	0.97
w_p	0.98	w_p	0.91
w_s	0.97	w_s	0.91

Moreover, we compared the parameter values estimated from the trial subset of the Jardri et al. study to our study's parameter values (Table E2). Interestingly, Mann-Whitney U tests showed increased weights in our sample (w_p, $p = 0.004$; w_s, $p = 0.002$).

Table E2. Means and standard deviations estimated from 130 trials of the Jardri et al. dataset compared to the current study.

	Jardri et al. CTL		Jardri et al. SCZ		This study	
**CII params**	μ	*SD*	μ	*SD*	μ	*SD*
a_p	0.196	0.176	0.133	0.194	0.180	0.174
a_s	0.041	0.035	0.250	0.216	0.084	0.120
w_p	0.361	0.107	0.232	0.242	0.470	0.199
w_s	0.580	0.158	0.775	0.139	0.697	0.194

It is important to remember that a quantitative comparison with the findings of Jardri et al. is limited by the fact that in the current study the diagnoses are self-reported, the number of trials reduced, and the study took place online, as opposed to a lab environment. Nonetheless, a qualitative comparison between our ASD group and the SCZ group of Jardri et al. shows a clear difference in sensory reverberation, with higher values in the SCZ patients, especially in the CII model (Fig E1).

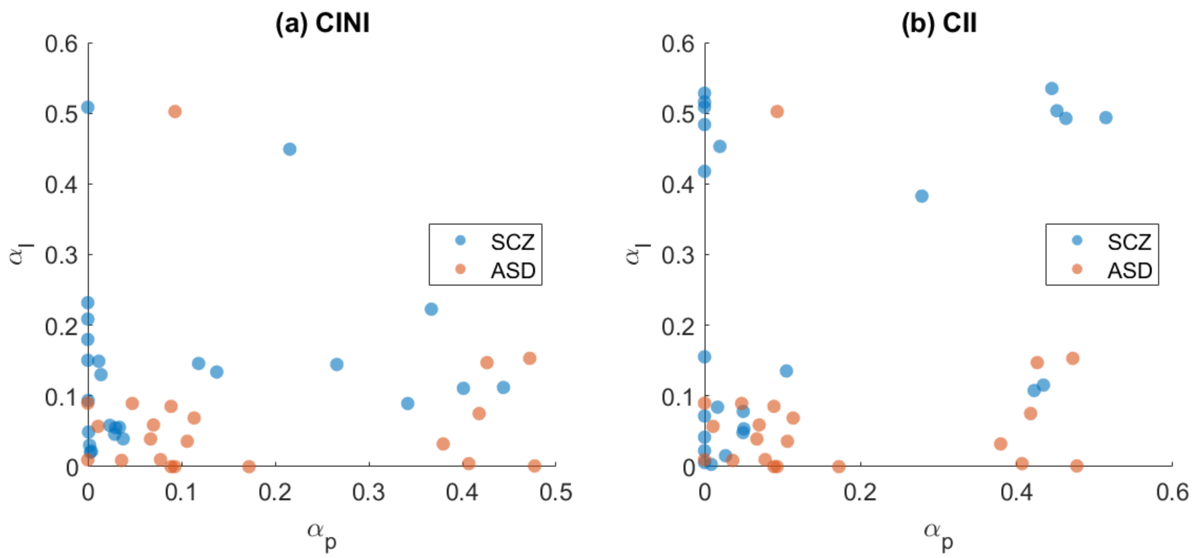

Fig E1. Reverberation parameters of the current study's ASD sample and Jardri et al.'s SCZ sample. *Each dot corresponds to one participant. SCZ parameter values were estimated using 130 trials of the Jardri et al. dataset.*

We also used the Jardri et al. dataset to more deeply investigate the comparisons between CII and CINI goodness of fit, in relation to trial set changes, parameter values, and psychiatric diagnoses and traits. The results from the Jardri et al. dataset showed that CII lost ground to CINI in the 130-trial set, and that patients are much better fitted by CII than CINI, while controls were slightly better fit by CINI (Table E3). Our study showed no difference between the ND and ASD groups, as both were clearly dominated by CINI.

Table E3. Fixed and random effects model comparisons in both studies.

Trialset	ΔBIC	*Pr*(CII)	ΔBIC	*Pr*(CII)	ΔBIC	*Pr*(CII)
**Jardri et al.**	Whole sample		CTL		SCZ	
**200**	192	0.61	–9	0.48	201	0.83
130	–27	0.53	–104	0.39	77	0.76
**This study**	Whole sample		ND		ASD	
130	–916	0.27	–291	0.31	–89	0.29

ΔBIC is the sum of individual CINI BIC scores minus individual CII BIC scores. Pr(CII) is the posterior model probability for CII, with Pr(CINI) = 1 – Pr(CII). CTL stands for control participants.

One possible explanation could be that the dominance of CII is associated with the size of the reverberation parameters. We tested that by looking at the Kendall correlations of CII reverberation

parameters with the individual ΔBIC = BIC(CINI) – BIC(CII). Interestingly, the results showed that this correlation only existed for the prior reverberation parameter and not the likelihood, and it appeared almost exclusively in patients with schizophrenia and not controls (Table E4).

Table E4. Kendall rank correlations between CII reverberation parameters and ΔBIC scores.

	**Jardri et al. CTL**		**Jardri et al. SCZ**		**This study**	
**CII params**	τ	p	τ	p	τ	p
$\boldsymbol{a_p, 200}$	0.31	0.03	0.63	< 0.001	–	–
$\boldsymbol{a_s, 200}$	0.22	0.13	0.19	0.19	–	–
$\boldsymbol{a_p, 130}$	0.08	0.59	0.53	< 0.001	–0.13	0.01
$\boldsymbol{a_s, 130}$	–0.13	0.39	0.17	0.24	–0.02	0.70

Overall, these results show that the dominance of CINI in our study, does not contradict the findings of Jardri et al. On the contrary, the results suggest that SCZ patients exhibit joint signal reverberation, in contrast with controls who independently overcount sensory or prior information.

References

1. Peer E, Brandimarte L, Samat S, Acquisti A. Beyond the Turk: Alternative platforms for crowdsourcing behavioral research. J Exp Soc Psychol. 2017 May;70:153–63.

2. Peirce J, MacAskill M. Building experiments in PsychoPy. Los Angeles: Sage; 2018. 297 p.

3. Bridges D, Pitiot A, MacAskill MR, Peirce JW. The timing mega-study: comparing a range of experiment generators, both lab-based and online. PeerJ. 2020 Jul 20;8:e9414.

4. Chmielewski M, Kucker SC. An MTurk Crisis? Shifts in Data Quality and the Impact on Study Results. Soc Psychol Personal Sci. 2020 May;11(4):464–73.

5. May JO, Looney SW. Sample Size Charts for Spearman and Kendall Coefficients. 2020;11:7.

6. Raftery AE. Bayesian Model Selection in Social Research. Sociol Methodol. 1995;25:111.

7. Jardri R, Duverne S, Litvinova AS, Denève S. Experimental evidence for circular inference in schizophrenia. Nat Commun. 2017 Apr;8(1):14218.

8. Ruzich E, Allison C, Smith P, Watson P, Auyeung B, Ring H, et al. Measuring autistic traits in the general population: a systematic review of the Autism-Spectrum Quotient (AQ) in a nonclinical population sample of 6,900 typical adult males and females. Mol Autism. 2015;6(1):2.

9. Frechner GT. Elemente der Psychophysik. Leipzig: Breitkopf und Härtel; 1860.
