# Supplementary material for: No increased circular inference in adults with high levels of autistic traits or autism
Source: PLoS Comput Biol. 2021 Sep 24;17(9):e1009006. doi: 10.1371/journal.pcbi.1009006 (PMC8494311; doi:10.1371/journal.pcbi.1009006)
Supplement: Attachment — Submitted filename: R2R.pdf [file pcbi.1009006.s003.pdf]

No increased circular inference in adults with high levels of autistic traits or autism: Reply to Reviewers

Nikitas Angeletos Chrysaitis[1], Renaud Jardri[23], Sophie Denève[2], and Peggy Seriès[1*]

We thank the reviewers for their time and thoughtful comments on our manuscript.
We have now made the recommended changes. Please find all reviewer comments in italics and our reply in normal font.

1. **Reviewer #1:**

Jardri & Denève (2013, Brain) proposed the circular inference theory—defective Bayesian inference in the brain—as a unifying account to explain many different symptoms in schizophrenia and autism. Computational models based on the circular inference theory had been tested in previous studies for schizophrenia patients versus healthy controls, whose results provide strong support for the theory. Using similar computational models, the current study provides the first empirical test of the circular inference theory on autism and autistic traits. They find that autism or autistic traits, different from schizophrenia, have little influences on human subjects' circular inference tendency.The technical quality of the present study is high and the paper is beautifully written. Though some of its results seem to contradict those of a previous study (Jardri et al., 2017, Nature Communications) that used the same task and a similar design (i.e., whether the data were better fit by the CII or CINI model), the authors' supplemental data analyses suggest that these differences can partly be explained by the slightly different experimental designs in the two studies.

We thank reviewer 1 for the positive comments.

The only issue that has raised my concern is the parameter recovery results reported in the Supplement for the two circular inference models (CINI and CII). As Figs. S2 and S3 show, when the simulated a_p is around 0.1, the recovered a_p does not follow a unimodal distribution centered at 0.1, but follows a bimodal distribution peaked at 0.1 and 0.4. Similarly, when the simulated a_p is 0.4, the recovered a_p follows a bimodal distribution peaked at 0.1 and 0.4. The situation is similar for a_s. It occurs to me that 0.1 and 0.4 may be two "attractors" for the estimated values of a_p and a_s. In other words, the values of a_p and a_s in the circular inference models may not be reliably estimated, at least from the current trial set.

This bimodal behaviour results from the fact that values greater than 0.15 for the a_p and a_s parameters have very similar effects on model predictions, as is evident from Fig R1 presented below. In this range, the data can be roughly approximated by treating prior information as binary ('left basket larger' vs 'right basket larger'), with very little dependency on the exact size of the baskets. We have added this explanation, along with figure R1 in the parameter recovery section of the supplementary information.

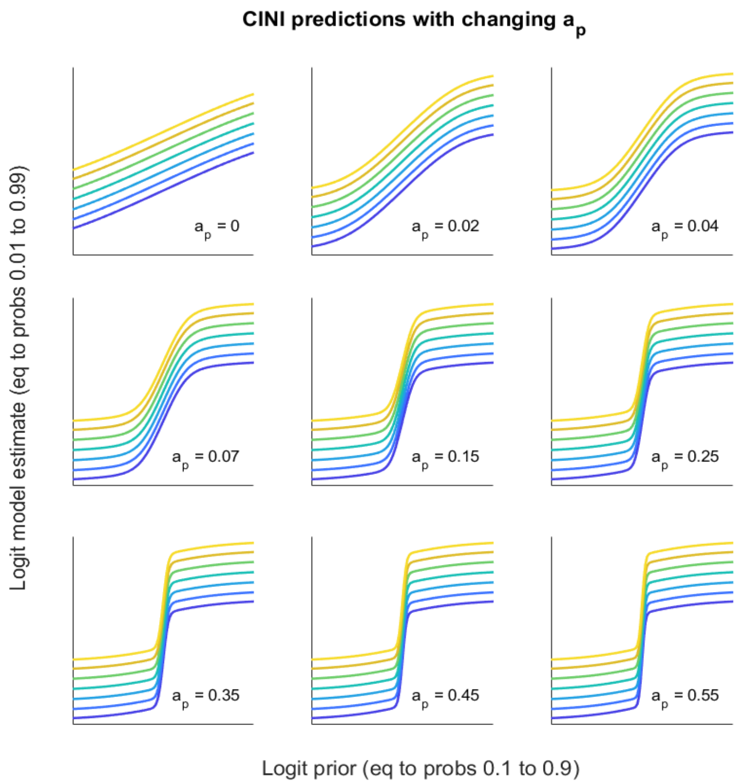

Fig R1. *Coloured lines correspond to different likelihood values (probabilities 0.1 to 0.9). Changes from a_p = 0 (top left) to a_p = 0.15 (middle) greatly affect observed model predictions, while those from a_p = 0.15 to a_p = 0.55 (bottom right) have almost no perceptible effect, despite the larger difference between parameter values.*

I am wondering whether the same bimodal-attractor issue would have occurred when the full set of 200 trials in Jardri et al., 2017 were used for parameter estimation. That is, whether the issue was closely associated with the circular inference models themselves, or only occurred for the subset of trials used in the present study. Is it possible for the authors to report the results of a parameter recovery analysis based on the full set of 200 trials? I understand that the results of this additional analysis are unlikely to change the conclusion, but would appreciate it given the light it may shed on future studies.

Parameter recovery in the full set of 200 trials showed that this bimodal pattern is present there as well (e.g., Fig R2 below). Moreover, the correlations between simulated and recovered parameters were very similar between trial sets (Table R1), with the maximum difference between Pearson coefficients being 0.71 for 130 trials vs 0.6 for 200, for a_s. We have added these results in the supplementary information (section B3, Fig B5 and Table B2).

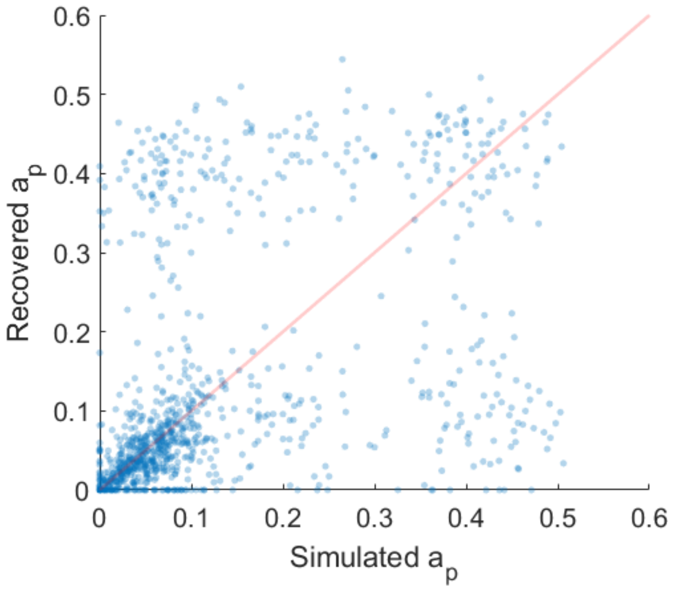

Fig R2. *Parameter recovery results for the prior reverberation in the full 200-trials set of Jardri et al. (2017). The red line corresponds to perfect recovery.*

	CINI		*CII*	
**Trials**	**130**	**200**	**130**	**200**
a_p	0.54	0.46	0.54	0.51
a_s	0.58	0.54	0.71	0.60
w_p	0.96	0.95	0.94	0.92
w_s	0.91	0.94	0.93	0.93

Table R1. *Pearson correlations between simulated and recovered parameters in the full 200-trials set of Jardri et al. (2017) and the current study.*

Minor:

1. One relevant study: Lu, Yi, and Zhang (2019, PLoS CB) investigated the influence of autistic traits on information sampling behaviors in the framework of Bayesian inference. Different from the findings of previous perceptual inference studies, they found little evidence that people with different autistic traits would differ in their weighting of likelihoods.

Lu, H., Yi, L., & Zhang, H. (2019). Autistic traits influence the strategic diversity of information sampling: Insights from two-stage decision models. PLoS Computational Biology, 15(12), e1006964. doi:10.1371/journal.pcbi.1006964

This is indeed a relevant study; we have added it to the discussion.

2. What are the neurobiological and psychological implications for the differences between the CII and CINI models?

Mathematically, both CII and CINI are simplifications of more detailed network models [see Jardri & Denève (2013) for the mathematical formulation]. As explained in the description of the models (page 10 of the main text), CINI assumes that the likelihood and the prior signals get reverberated and overcounted separately and are only combined after they have been reverberated, while in CII they are combined, and they interfere with each other during the reverberation.

We can only speculate about the possible neurobiological underpinnings of those models. However, the detailed network models suggest that the reverberation and amplification of both signals could be related to imperfect inhibition leading to increased E/I ratio. In the present participants sample, the imbalance might be weak or localized, affecting the signals only separately. In patients with schizophrenia, however, for whom CII is a better fit, the imbalance might be larger and extend across the cognitive hierarchy, which would lead to interference between priors and likelihoods.

At a functional level, in both CINI and CII, the reverberation of each signal would lead to overconfidence. However, when prior or likelihood signals are relatively certain (probabilities close to 0 or 1), the two models differ (see e.g., Fig 2 in the main text). Specifically, CINI is symmetrical in how it treats the combination of the two signals when they agree vs when they do not. For example, changing the prior from 0.7 to 0.8 or from 0.3 to 0.2 moves the confidence estimate an equal amount (albeit in opposite directions). On the other hand, in CII, this depends on the likelihood value. If the likelihood has a value of 0.2, then a prior change of 0.3 to 0.2 has only a marginal effect on confidence, as the model confidence estimate is already very close to 0. Changing the prior from 0.7 to 0.8, though, has a very large impact (for example, see Table R2 below). This essentially means that a person who is better approximated by CINI still maintains some uncertainty in their confidence estimates, despite their overconfidence. On the other hand, a person who is better approximated by CII will exhibit a high degree of certainty based on only one signal (which one it is will depend on the a_p and a_s values) and is only moved from that estimate when the other signal has strong,contradicting information.

We have mentioned in the Discussion that CINI might be expressing the separate reverberation of priors and likelihoods in the brain, while in CII they are reverberated together. We have expanded upon this discussion:

'The dominance of CINI across our sample (Table E3 in S1 Supplementary Information) is an indication that prior beliefs and sensory evidence are reverberated, even in healthy participants. While we can only speculate about the possible neurobiological underpinnings of our circular inference models, we proposed that reverberation arises due to an increased E/I ratio, based on the network model of Jardri & Denève [23]. If that is the case, CINI might correspond to a weak or localised E/I imbalance, affecting the signals only separately. In schizophrenia, then, this

imbalance would be larger and extend across the cognitive hierarchy, which would lead to interference between priors and likelihoods, making CII the better fit for these participants.’

Jardri, R., & Denève, S. (2013). Circular inferences in schizophrenia. Brain, 136(11), 3227-3241.

Prior	*SB*	*WB*	*CINI*	*CII*
**0.2**	-2.77	-2.59	-6.65	-7.29
**0.3**	-2.23	-2.1	-5.98	-7.22
**0.7**	-0.54	-0.49	-0.67	-2.16
**0.8**	0	0	0	0

Table R2. *Model confidence estimates (logits) with different prior values (probabilities). All confidence estimates assume a likelihood value of 0.2. WB, CII, and CINI used w_p = w_l = 0.95. CI and CINI used a_p = a_l = 0.05. In CII, a prior value change from 0.3 to 0.2 moves the model estimate only by -0.09, while a change from 0.7 to 0.8 moves it by 2.16.*

2. **Reviewer #2:**

This paper examines the relationship between ASD and reverberation of prior and sensory evidence in a decision-making task. It leverages previous findings of signal reverberation in patients with schizophrenia and the notion that ASD and schizophrenia share numerous similarities. While the group of ASD patients is small and the self-reported diagnoses are unverified, they also tested a larger sample to examine correlations with ASD traits in a typical population. Contrary to expectations, the authors did not find any evidence of signal reverberation in individuals with ASD and no correlation with ASD traits. Despite the somewhat inconclusive results given the unverified patient sample, the topic is timely. The paper is exceptionally well written, concepts are explained clearly and in an accessible manner. I also appreciate the rigorous methodological approach, including the use of Bayesian statistics to support the null result.

We thank reviewer 2 for the positive comments.

I have some comments about the interpretation of the findings, especially of the w parameter. In addition, some issues with the task design make the results somewhat less conclusive.

- *In the abstract, please clarify that the ASD diagnosis was self-reported. This especially seems important since the AQ score of the group was lower than previously reported, suggesting that the sample was not very representative.*

We agree that this is important, and we have changed the abstract and parts of the main text accordingly.

● *It is not certain that the fish baskets can be interpreted as priors and the lakes as sensory information. As the authors also state in the discussion, the task might also be interpreted as a delayed cue integration task. Moreover, I'm not sure if people are good at estimating the relative size of objects (the fish baskets), if it is linear, and if it is comparable to estimation of relative frequency (the fish in the lakes). While indeed this does not change the results, it does make the interpretation of the results less conclusive.*

We agree that there are other possible interpretations of the results, as we have mentioned in the discussion. This task was chosen because it was the one used before, for which behaviour had been described to be consistent with the circular inference model and where differences have been found in patients with schizophrenia (Jardri et al., 2017).

For the estimation of the basket size, we have tested 4 additional models that estimated basket sizes in different ways. All of them are based on *CINI*, but with some additional modifications for the basket sizes. The first model rescaled the perceived probabilities linearly:

$$q_i = k\,(p_i - 0.5) + 0.5, \quad i \in \{l, r\}, k \in (0, 1),$$

with *p* corresponding to the intended probabilities, *q* to the perceived ones, *i* to the left or right basket, and *k* to the rescaling factor. The second model rescaled them exponentially:

$$q_i = {p_i}^k \,/\, ({p_l}^k + {p_r}^k), \quad i \in \{l, r\}, k \in (0, +\infty)\ .$$

The third was based on the Weber-Frechner law, where perceived sizes would be proportional to the logarithm of the actual sizes:

$$q_i = ln\,(p_i \,/\, p_0) \,/\, [ln\,(p_l \,/\, p_0) + ln\,(p_r \,/\, p_0)], \;\; p_0 \in (0, 0.1),$$

where p0 corresponds to the just noticeable basket size. Finally, the fourth model treated basket sizes as providing only binary information ('left basket larger' vs 'right basket larger'), which nudged the fish frequency-based estimates by a constant amount. Then the logit confidence becomes:

$$L_c = F(L_s, w_s) \pm A, \;\; A \in (-\infty, +\infty)\ ,$$

with *A* being the constant amount, positive when the left basket was larger than the right, negative in the opposite case, and equal to 0 when sizes were equal.

The first three models had 5 parameters each, while the last one had only 3. Both fixed and random effects comparisons with *CINI* showed clear superiority for *CINI* (group *Δ*BIC > 715, posterior model probability for *CINI* > 0.72). This information has been added to S1 Supplementary Information, section D4.

● *The fish baskets are presented before the lakes, with a blank screen in between. Because of this sequential nature of the task, could one also interpret the findings in patients with schizophrenia as a bias to rely on more recently observed evidence? It seems like a potential confound in these studies, partly because working memory can be impaired in patients or inversely correlated with AQ.*

We edited our discussion to include a reply to these concerns:

'In the fisher task, the baskets are presented before the lakes. This means that participants might simply display a recency bias, where the most recent evidence is overweighted. Under the Bayesian framework, the earlier evidence should create a prior belief in the participants, which is then combined with and updated by following evidence. Therefore, a recency bias is indistinguishable from an overweighting of sensory evidence. A possible issue, though, is that behavioural differences might be related to differences in the working memory of the participants. This could be especially important, since working memory is impaired in both ASD and schizophrenia (Wang et al., 2017; Forbes et al. 2009). However, Jardri et al. (2017) measured working memory performance in their sample and showed that it is correlated only with the prior weights, but not with the reverberation parameters. This would need to be validated in further studies, but we therefore expect that our findings regarding circular inference in autism should be robust to potential differences in working memory.'

Wang, Y., Zhang, Y. B., Liu, L. L., Cui, J. F., Wang, J., Shum, D. H., ... & Chan, R. C. (2017). A meta-analysis of working memory impairments in autism spectrum disorders. Neuropsychology review, 27(1), 46-61.

Forbes, N. F., Carrick, L. A., McIntosh, A. M., & Lawrie, S. M. (2009). Working memory in schizophrenia: a meta-analysis. Psychological medicine, 39(6), 889.

"Due to concerns about participants' potential distractibility in an online environment if the task was too long, we reduced the number of trials to 130." Were 130 trials also used for the parameter identification analyses? The effect of reducing the trial number is explained for model recovery, but the parameter recovery should also give an indication of whether 130 is a sufficient amount.

The 130 trials were indeed used for the parameter recovery. Please refer to Table R1 in our reply to reviewer 1, which shows very similar recovery for 130 vs 200 trials. We have included this information in the supplementary information and clarified that the parameter recovery results were for the 130 trials in the main text.

● *In the methods, the model free analyses section doesn't clarify whether any interaction effects were included in the model and whether any non-linear terms were added. Please also clarify whether any variants of the mixed-effects models (e.g., with interaction terms or non-linear effects) were tested and compared. Perhaps this can be added to the supplement.*

Initially we had used only one linear mixed-effects model, which did not include any interactions effects or non-linear terms. We have now expanded our analysis. The Methods section now reads:

‘Linear mixed-effects models (LMEs) were used to verify that participants combined the information of both baskets and fish ratios when making their decisions and to investigate any possible interactions with autistic traits. We chose the absolute confidence of the participants as the response variable (|*c* – 0.5|, with c being the participant confidence estimate). We modelled the following as fixed effects with repeated measures across the subjects, in all LMEs: i) the absolute likelihood (|*likelihood* – 0.5|); ii) the prior congruency, that is how much the prior agreed with the likelihood (|*prior* – 0.5| * sgn[(*prior* – 0.5*)(likelihood* – 0.5)]); iii) the reaction times. All LMEs also included the two-way interaction between i and ii, with iii being used to investigate the possibility of a speed-accuracy trade-off, and the participants being treated as a random factor. We analysed our results with 5 different LME variants. The first one, LME_core only used the aforementioned components. LME_AQ expanded upon LME_core by including a fixed effect for AQ and the two- and three-way interactions of AQ with i and ii. LME_PDI was the same as LME_AQ but with the PDI scores instead of the AQ. Then, LME_full, used both AQ and PDI and their interactions with i and ii, but no interactions between them. Finally, LME_rtInteract expanded upon the LME_full to include interactions between AQ or PDI and reaction times. Full specification of the models in Wilkinson notation can be found in Section B1 of S1 Supplementary Information.’

Accordingly, the corresponding Results section now reads:

‘Among the linear mixed-effects models, the one which achieved the smallest BIC was LME_core (ΔBIC: LME_PDI, 17; LME_AQ, 35; LME_full, 51; LME_rtInteract, 69). All models confirmed the influence of both absolute likelihood (e.g., LME_core: t=44.50, p<10^–323) and prior congruency (e.g., LME_core: t=24.63, p=10^–132), as well as the interaction of the two components (e.g., LME_core: t=25.20, p=10^–138). Despite the LME_core being the best model, both LME_PDI and LME_full showed significant association between absolute confidence and non-clinical delusional beliefs (PDI) (e.g., LME_PDI results: t=2.08, p=0.037) and an interaction between absolute likelihood and PDI (e.g., LME_PDI results: t=2.31, p=0.021). However, neither the influence of autistic traits (AQ) or its interactions with model components were significant in the LME_AQ and LME_full models. Reaction times showed a negative relationship with absolute confidence in all models (e.g., LME_core: t= –17.01, p=10^–64), which is presumably a result of participants taking more time to respond when they are uncertain [51]. Importantly though, the LME_rtInteract achieved the worst BIC score, with no interaction between psychiatric traits and reaction times (LME_rtInteract: PDI t=1.29, p=0.20; AQ t=0.72, p=0.47). This suggests that any possible relationship between AQ or PDI and participant behaviour is not a result of differences in time management. The full LME results can be found in Section B1 of S1 Supplementary Information.’

- *The equation for the sigmoid is given as:* $F(L,w) = ln([we^{L} + 1 - w]/[(1-w)e^{L} + w])$

In the text it’s stated that “a weight value of w=0 shows no influence of the corresponding signal”. Perhaps I’m misunderstanding something but that appears to be incorrect? In the sigmoid equation, if w = 0, then the equation reduces to: $F(L,0) = -L$
In other words, the corresponding signal is completely inverted at w =0. For the task, I think this

means that the agent would assign the signal (i.e., evidence) to the wrong lake, as we're working with logits and the likelihoods sum to 1.

Following the provided sigmoid equation, the signal has no influence at w = 0.5 because then L = 0. If w < 0.5 this means that the agent starts to invert the signal. When we look at figure 6, there is some data at w < 0.5. If I'm correct then this changes the interpretation of the weights data.

It is true that, following the equations, a signal would have no influence at w=0.5, rather than at w=0. The confusion here comes from the fact that all the weight values reported were rescaled from the [0.5, 1] range to [0, 1]. This was mentioned in the supplementary information, but we have now moved this section to the methods:

'True parameter ranges were [0.5, 1] for the weights (w) and [0, 60] for the reverberation parameters (a), however, these were rescaled to [0, 1] so that they could be easily compared with those reported by Jardri et al. 60 is an arbitrary upper limit, that however is high enough for our purposes, as no parameters approached it (max non-rescaled CINI a = 29.02). In the rest of this article, we will be referring exclusively to the rescaled parameters, however the word 'rescaled' will be omitted for conciseness.'

- *Given that the paper's main hypothesis was that reverberation would be higher in people with ASD (traits), I was a bit surprised to see that the parameter recovery simulations mostly made use of low reverberation parameter values. The reverberation a p parameter in both the CINI and the CII model is less identifiable at values >0.15 (supplement). This seems to be true for a_s also, but even fewer data points are simulated at higher a s . This is more problematic for a_p as figure 6 and the supplement suggest that the model returned a_p > 0.15 for some participants. Fortunately it is less an issue for a_s as more data seem to fall in a lower range. Perhaps suggestions could be made on how to improve the identifiability of high reverberation values in future research.*

We decided to base our simulations for parameter recovery on the estimated parameters from our models, as explained in Section A6 of the Supplementary Information (Section B3 in the updated SI). It is true that values higher than 0.15 for parameters a_p and a_s are less identifiable, and therefore we believe that fitting the whole [0, 1] range would give misleading estimates for the identifiability of the participant parameters. We believe that the identifiability problems occur because values greater than 0.15 have very similar effects on model predictions; please see our first answer to reviewer 1, including Fig R1.

Minor comments:

"which they could repeat many times before proceeding to the task" this sentence seems to be about something else than the instruction to be as fast and accurate.

We have changed the syntax to make it clearer: 'Participants were presented with detailed instructions which they could view many times before proceeding to the task. The instructions made clear that participants should respond "as fast and as accurately as possible".'

Please number the equations in the main text.

The equations have been numbered.

"Each weight w expresses the reliability of the corresponding signal." Reliability or subjective weight?

We agree that the weights express only an estimate of the signal reliability, which will be subjective and different for each participant. We have changed the text to clarify what the weights represent: 'Each weight *w* determines the influence of the corresponding signal to the confidence estimate. This depends on how the reliability of that signal is estimated by each participant.'

3. **Reviewer #3:**

General comments

I think that this manuscript is a valuable contribution to the fields of bayesian accounts of autism and, more broadly, computational psychiatry. The empirical and computational methods were presented clearly and the code was documented in detail.

We thank reviewer #3 for the positive comments

I would like to raise the following general comments about the approach: The paper draws on earlier computational research in perception in autism (references [55-58]) and suggests that the contrast between the current data and these studies could be related to the use of decision-making rather than perceptual tasks. In my opinion, two points need to be considered. The most important difference is that in some of these studies limitations related to prior knowledge could be due to aberrant mechanisms in extracting prior knowledge statistics. In this task, prior knowledge is not formed across trials, but is instead is presented in each trial. It is as if participants are asked to mentalise, "suppose you have this knowledge. What would you decide?"- rather than experience the knowledge themselves. There is a substantial perception element (size or numerosity) influencing the interpretation of priors and likelihoods with baskets and ponds.

We agree that this is an important point and we have changed the discussion to mention this difference between our experiment and most of the relevant literature:

'However, these effects have been demonstrated exclusively in perceptual tasks, with the rare study of Bayesian decision-making in ASD showing no such imbalance [59, 60]. Another important difference is that in most of the literature, participants have to learn prior beliefs based on the observed statistics, while in our study they are explicitly presented by the size of the baskets. It is possible that the cause of the prior-likelihood imbalance found in the literature lies in impaired prior acquisition, rather than in the relative weighting of the prior per se.'

The unexpected patterns of not-so-high AQ in the autistic group is puzzling and possibly worrying. Overall, the authors provide good detail on how they mitigated potential limitations of requirement and testing via online platforms (I suspect as an alternative given covid-19?). For example, it was useful to include attentional controls in the task. Similarly, I think the authors needed to include a second measure of autistic symptomatology, e.g., SRS-2. to corroborate the autism diagnosis. This is deemed as necessary in studies that involve face-to-face testing, and more so it would be very important in an online setting.

Corsello C, Hus V, Pickles A, Risi S, Cook EH Jr, Leventhal BL, Lord C. Between a ROC and a hard place: decision making and making decisions about using the SCQ. J Child Psychol Psychiatry. 2007 Sep;48(9):932-40. doi: 10.1111/j.1469-7610.2007.01762.x. PMID: 17714378.

The use of an online platform was indeed partly motivated by covid-19. We agree that such studies should be repeated in a sample where diagnoses are verified by a clinical professional, as mentioned in the discussion. However, we believe that the AQ results are reliable, as the questionnaire had high internal consistency in our sample [Cronbach's a = 0.76, higher than the value of a = 0.67 which has been previously reported in the literature (Hurst et al. 2007)] and all participants correctly responded to both attention checks. We have slightly edited our paper to shift its focus to autistic traits, instead of diagnoses.

Hurst, R. M., Mitchell, J. T., Kimbrel, N. A., Kwapil, T. K., & Nelson-Gray, R. O. (2007). Examination of the reliability and factor structure of the Autism Spectrum Quotient (AQ) in a non-clinical sample. Personality and Individual Differences, 43(7), 1938-1949.

Another major limitation is the lack of cognitive measures. In the original study on scz, the two groups were closely matched with each other in neuropsychological evaluation measures. I think having a measure of performance and verbal intelligence was relevant here. In terms of methodological rigour, this could ensure that any potential group differences are not due to differences in perceptual or verbal reasoning abilities, but related to autism symptomatology. These measures are important as this task relies on perceptual and verbal reasoning abilities.

We agree that the addition of cognitive measures would strengthen the results of our study. The choice not to include them was based on concerns revolving around the experiment duration (which already lasted 30 minutes) in an online environment, and the potential distractibility of the participants.

It is true that group differences might have been a product of different perceptual or verbal abilities. However, the majority of our sample did not have a diagnosis of any mental disorder, and autistic trait scores showed no relationship with participant behaviour. We have changed the discussion to mention that future studies would benefit from including such measures. The text now reads:

'Our findings will need to be confirmed in a sample verified by a mental health professional, especially as the criteria for an ASD diagnosis have largely changed between versions of the Diagnostic and Statistical Manual of Mental Disorders [61]. Such a study would also benefit from

cognitive measures, to ensure that perceptual or verbal reasoning abilities do not constitute a confounder for any differences between the groups.'

I think that the authors need to make a clearer decision on whether they situate this study as a cross-syndrome comparison of asd and scz with neurotypicals as reference or whether they compare asd to nt with reference to a relevant earlier scz study. If the former, I think that a direct comparison with the findings with the scz individuals is missing. There was a stark contrast between scz and nt performance in the original study, and no difference at all in this study. This message should be conveyed more clearly. Some considerations are given in supplementary info, a plot would be needed too.

Our study's main focus is on the relationship between autistic traits or the autism spectrum and "circularity" in Bayesian inference that would be suspected to arise from E/I imbalances observed in this disorder. The comparison between ASD and SCZ was one of our motivations and we find it important to discuss it as well.

A quantitative comparison with the findings of Jardri et al. 2017 is however limited by the fact that here the diagnoses are self-reported, the number of trials is reduced, and the study took place online, as opposed to a lab environment. We have changed the introduction and discussion to clarify the focus of the current study: 1) ASD vs NT, and 2) (at a more qualitative level) ASD vs SCZ. We have also added the plot below as a qualitative comparison between the conditions (Fig E1 in S1 SI), which shows higher likelihood reverberation in SCZ patients, compared to ASD individuals.

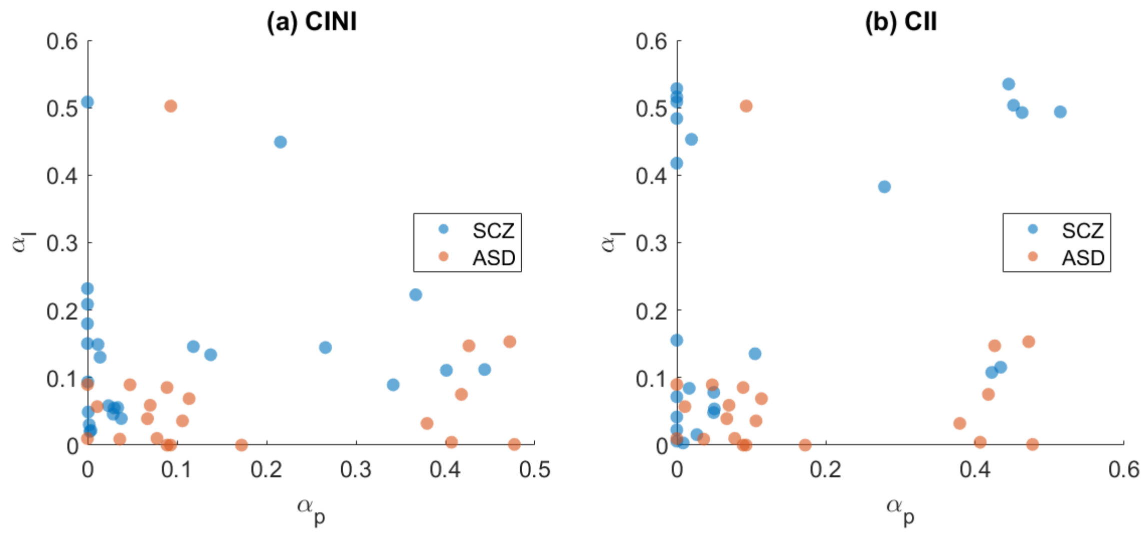

Fig E1. Reverberation parameters of the current study's ASD sample and Jardri et al.'s SCZ sample. *Each dot corresponds to one participant. SCZ parameter values were estimated using 130 trials of the Jardri et al. dataset.*

Minor comments:

p.8 Interestingly, the participants with the ASD diagnoses had AQ scores on the low-end (M=28.0, 146 SD=8.0) compared to those reported in the literature for autistic individuals (M=35.2, SD=6.3) [43]

- What was the gender distribution in the autism group?

What do the authors make of it? Is it limitations of AQ or limitations of online recruitment or chance? This needs to be extended.

The gender distribution of the ASD group was 13 M and 8 F, similar to the one in the general sample. We have included the gender distribution of all groups in the text. We have added the following line in the discussion: 'Moreover, the diagnoses of our participants in the Prolific subsample were self-reported. What those diagnoses were based on, and when they were delivered is uncertain, which could explain the atypical AQ scores of the ASD group.'

-line 275 to outliers [50] (Fig A2 and A3 in S1 Supplementary Information).S2 S3?

-and line 277 also showed no correlation between different parameters (Table A3 and A4 in S1...: It is S3 and S4?

The supplementary figures and tables are named starting with the letter of their corresponding section in S1 Supplementary Information. We did not choose names beginning with S so that they are not confused for additional supplementary files, instead of part of S1 SI.

- I think that a discussion paragraph outlining the similarities of accounts of autism and schizophrenia would be a useful addition.

We have changed the discussion to outline the similarities between the conditions. The text now reads:

'On that account, the absence of any difference between our participant groups is surprising, given the observed inhibitory impairments in ASD [33,34] and the commonalities between autism and schizophrenia regarding E/I imbalances [53,54]. Moreover, prominent computational explanations for the two conditions suggest that they share similar Bayesian impairments. Specifically, it has been proposed that an imbalance of likelihoods to priors, in favour of the former, lies at the heart of both ASD and SCZ [12-16]. This seems to be contradicted by our findings, which showed no increase in reverberation along the autism spectrum, despite the presence of such an increase in schizophrenia. This is further exhibited in a qualitative comparison between the conditions, which showed higher likelihood reverberation in SCZ (Fig E1 in S1 Supplementary Information).'

Have the authors made all data and (if applicable) computational code underlying the findings in their manuscript fully available?

Reviewer #1: Yes

Reviewer #2: None

Reviewer #3: Yes

As reviewers #1 and #3 have identified, we have included a link to code and data in the Methods section (https://osf.io/yqug2/), lines 250-251 in the previous version and line 293 in the current one.
