# Supplementary material for: No increased circular inference in adults with high levels of autistic traits or autism
Source: PLoS Comput Biol. 2021 Sep 24;17(9):e1009006. doi: 10.1371/journal.pcbi.1009006 (PMC8494311; doi:10.1371/journal.pcbi.1009006)
Supplement: Attachment — Submitted filename: Review comments.docx [file pcbi.1009006.s002.docx]

This paper examines the relationship between ASD and reverberation of prior and sensory evidence in a decision-making task. It leverages previous findings of signal reverberation in patients with schizophrenia and the notion that ASD and schizophrenia share numerous similarities. While the group of ASD patients is small and the self-reported diagnoses are unverified, they also tested a larger sample to examine correlations with ASD traits in a typical population. Contrary to expectations, the authors did not find any evidence of signal reverberation in individuals with ASD and no correlation with ASD traits. Despite the somewhat inconclusive results given the unverified patient sample, the topic is timely. The paper is exceptionally well written, concepts are explained clearly and in an accessible manner. I also appreciate the rigorous methodological approach, including the use of Bayesian statistics to support the null result. I have some comments about the interpretation of the findings, especially of the *w* parameter. In addition, some issues with the task design make the results somewhat less conclusive.

- In the abstract, please clarify that the ASD diagnosis was self-reported. This especially seems important since the AQ score of the group was lower than previously reported, suggesting that the sample was not very representative.
- It is not certain that the fish baskets can be interpreted as priors and the lakes as sensory information. As the authors also state in the discussion, the task might also be interpreted as a delayed cue integration task. Moreover, I’m not sure if people are good at estimating the relative size of objects (the fish baskets), if it is linear, and if it is comparable to estimation of relative frequency (the fish in the lakes). While indeed this does not change the results, it does make the interpretation of the results less conclusive.
- The fish baskets are presented before the lakes, with a blank screen in between. Because of this sequential nature of the task, could one also interpret the findings in patients with schizophrenia as a bias to rely on more recently observed evidence? It seems like a potential confound in these studies, partly because working memory can be impaired in patients or inversely correlated with AQ.
- “*Due to concerns about participants' potential distractibility in an online environment if the task was too long, we reduced the number of trials to 130.”*

Were 130 trials also used for the parameter identification analyses? The effect of reducing the trial number is explained for model recovery, but the parameter recovery should also give an indication of whether 130 is a sufficient amount.

- In the methods, the model free analyses section doesn’t clarify whether any interaction effects were included in the model and whether any non-linear terms were added. Please also clarify whether any variants of the mixed-effects models (e.g., with interaction terms or non-linear effects) were tested and compared. Perhaps this can be added to the supplement.
- The equation for the sigmoid is given as:

In the text it’s stated that “a weight value of w=0 shows no influence of the corresponding signal”. Perhaps I’m misunderstanding something but that appears to be incorrect? In the sigmoid equation, if w = 0, then the equation reduces to:

$F\left( L,w \right)=\ln\left( \frac{1}{e^{L}} \right)= -L$

In other words, the corresponding signal is completely **inverted** at w =0. For the task, I think this means that the agent would assign the signal (i.e., evidence) to the wrong lake, as we’re working with logits and the likelihoods sum to 1.

Following the provided sigmoid equation, the signal has no influence at w = 0.5 because then L = 0. If w < 0.5 this means that the agent starts to invert the signal. When we look at figure 6, there is some data at w < 0.5. If I’m correct then this changes the interpretation of the weights data.

- Given that the paper’s main hypothesis was that reverberation would be higher in people with ASD (traits), I was a bit surprised to see that the parameter recovery simulations mostly made use of low reverberation parameter values. The reverberation *a_p_* parameter in both the CINI and the CII model is less identifiable at values > 0.15 (supplement). This seems to be true for *a_s_* also, but even fewer data points are simulated at higher *a_s_*. This is more problematic for *a_p_* as figure 6 and the supplement suggest that the model returned *a_p_* > 0.15 for some participants. Fortunately it is less an issue for *a_s_* as more data seem to fall in a lower range. Perhaps suggestions could be made on how to improve the identifiability of high reverberation values in future research.

Minor comments:

*“which they could repeat many times before proceeding to the task”* this sentence seems to be about something else than the instruction to be as fast and accurate.

Please number the equations in the main text.

“Each weight w expresses the reliability of the corresponding signal.” Reliability or subjective weight?
